# Concomitant primary hyperparathyroidism and systemic lupus erythematosus: coincidence or not? A new case report

**DOI:** 10.11604/pamj.2020.37.228.26257

**Published:** 2020-11-11

**Authors:** Habiba Bennesser Alaoui, Siham Hamaz, Houda Bachir, Ahmed Amine Eloumri, Mohammed Berrimi, Abdellatif Bouayad, Khalid Serraj

**Affiliations:** 1 Department of Internal Medicine, Mohammed VI University Hospital of Oujda, Oujda, Morocco,; 2 Immunohematology and Cellular Therapy Laboratory, Medical School of Oujda, Mohammed First University of Oujda, Oujda, Morocco

**Keywords:** Hypercalcemia, primary hyperparathyroidism, systemic lupus erythematosus, case report

## Abstract

Primary hyperparathyroidism (PHP) is the most common cause of hypercalcemia. Patients with systemic lupus erythematosus (SLE) can develop hypercalcemia but it is exceptionally due to PHP. There are only few cases of concurrent SLE and primary hyperparathyroidism (PHP) described in the literature. We report a case of a 31-year-old patient having SLE with lupus nephritis class III and anti-phospholipid syndrome, complicated by pulmonary embolism associated to primary hyperparathyroidism causing severe hypercalcemia and osteoporosis. Even if there is no evidence for potential pathogenic association between PHP and SLE, the recognition of this association is very important because of therapeutic and prognostic impact. Early detection of PHP leads to avoid severe complications and significant morbidity.

## Introduction

Primary hyperparathyroidism (PHP) is a metabolic disease resulting from inappropriate and excessive production of parathyroid hormone (PTH). It is the most common causes of hypercalcemia. Patients with systemic lupus erythematosus (SLE) can develop hypercalcemia but it is exceptionally due to PHP [1]. There are only few cases of concurrent SLE and primary hyperparathyroidism (PHP) described in the literature. We report a new case.

## Patient and observation

A 31-year-old woman with no pathological history was admitted to our hospital for chest pain, bone and joint pain, hair loss and asthenia for the last 3 months. On physical examination, we found fever at 38°C, polypnea at 32 cycles/min, arterial hypertension at 160/90 mmHg, malar rash, synovitis of wrists, elbows and ankles, and edema on both legs.

**Figure 1:**
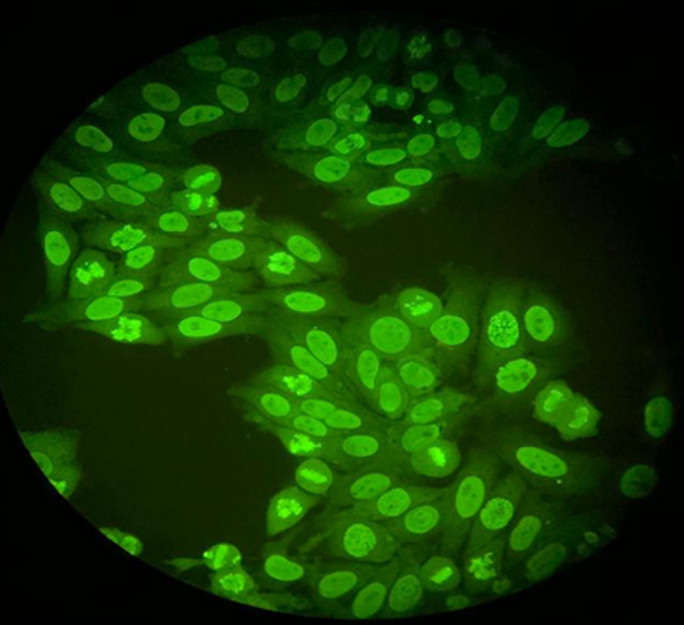
homogeneous pattern of antinuclear antibody (ANA)

**Figure 2:**
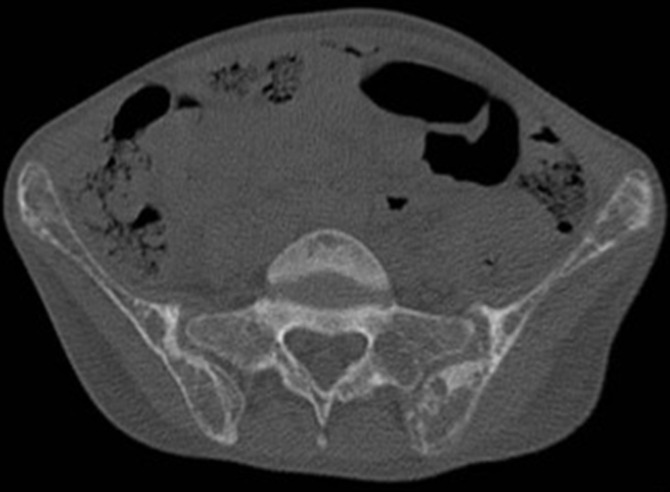
pelvic osteolytic lesions

**Diagnostic assessment:** laboratory tests disclosed the following values: Increase level of erythrocyte sedimentation rate 120 mm/1^st^ hour, C reactive protein was 20 mg/l. The blood count showed hemoglobin at 9g/dl, MCV at 85 µ^3^, lymphopenia at 400/mm^3^, while platelets were normal 450 000/mm^3^. Direct coombs test was negative. Serum Albumin was 25g/l; proteins level was low at 50g/l and proteinuria at 3.2g/day. Creatinine was normal. Anti-nuclear antibody ANA were positive 1/320 with homogeneous pattern (Figure 1), anti-dsDNA level was 160U/ml. Anti-cardiolipin IgG antibodies were positive 25UGPL. Rheumatoid Factor was negative. C3 and C4 complement fractions were low (0.2 g/l and 0.05 g/l). Chest x-ray, electrocardiogram and echocardiography were normal. Chest computed tomography (CT) scan showed proximal pulmonary embolism. Renal ultrasound was normal. Renal biopsy reveals immunocomplex nephritis, lupus nephritis, segmental mesangial proliferation, mild activity lupus nephritis class III (A/C). Before starting corticosteroids, we analyzed electrolytes. Serum calcium was elevated 132 mg/l with hyper-calciuria 479 mg/24 h. 25 OHD was normal. Alcaline phosphatase was 420 UI/l. the patient did not have any symptoms of hypercalcemia. Serum protein Electrophoresis showed polyclonal hypergamma globulinaemia. Serum and urine immunofixation as well as Bence-Jones proteinuria were negative. Intact parathyroid hormone (iPTH) was high 628 pg/ml. CT scan and ultrasound of parathyroid imaging revealed a lower left parathyroid nodule measuring 2cm x 1 cm. Femoral and lumbar bone mineral density (BMD) showed osteoporosis (T-score: -2.6). In addition, we found multiple pelvic osteolytic lesions at CT scan (Figure 2). Other causes of hypercalcemia and bone lysis were excluded. The diagnosis in this case was SLE with lupus nephritis class III and anti-phospholipid syndrome, complicated by pulmonary embolism associated to primary hyperparathyroidism causing severe hypercalcemia, osteoporosis.

**Therapeutic intervention:** the patient was given a pulse of methyl-prednisolone 15 mg/kg/day for 3 days followed by oral prednisone 1 mg/kg/day, associated with intravenous cyclophosphamide 750 mg/m^2^/month for 6 months. Mycophenolate mofetil 2 g/day was prescribed as a maintenance therapy of lupus nephritis with hydroxychloroquine at a dose of 400 mg/day. Treatment of pulmonary embolism was initiated with subcutaneous low molecular weight heparin (enoxaparin 0.1 ml/10 kg/12h) followed by antivitamin K. For Hypercalcemia, patient has received intravenous fluid with furosemide. She also required Alendronate 70 mg/week with Vitamin D 400u/day for osteoporosis. The left parathyroid gland was surgically removed. Histopathological examination revealed parathyroid adenoma. Her immediate postoperative parathyroid hormone level was 64 pg/ml with a calcium level of 98 mg/l.

**Follow-up and outcomes:** forty-eight hours after the surgery she developed oral paresthesia. She had hypocalcemia at 72 mg/l. She required oral supplementation for few months. Additional investigations for multiple endocrine neoplasia were negative. The patient remained asymptomatic. Her SLE was calm without any relapse. Control of proteinuria was negative. Corticosteroids was dropped. The follow-up was 4 years.

## Discussion

Primary hyperparathyroidism is a metabolic disorder resulting from excessive production of parathyroid hormone (PTH) and is the most common cause of hypercalcemia in the general population [1]. It can occur at any age but the majority of cases occur around 40 to 50 years old. Women are affected two to three times more than men [2]. Approximately 80% to 85% of patients with PHP have parathyroid adenoma [3]. Other causes of hypercalcemia are represented mainly by malignancy, multiple myeloma or a lymphoproliferative disease. Rarely, hypervitaminosis D, sarcoidosis, other granulomatous diseases, some drugs and endocrine diseases with increased bone turnover may be responsible [3]. The association of systemic lupus erythematosus and hypercalcemia is rare. To our knowledge, only 18 cases of patients with SLE with hypercalcemia, have been previously reported in a recent literature review [3]. Mechanisms by which hypercalcemia may occur in patients with SLE have been only partially clarified. The possible pathogenesis involves elevated blood PTH level, excessive endogenous production of PTH related peptide (rP) or the presence of autoantibodies against PTH receptor antibodies [2,4-6]. PTHrP may be produced by non-malignant lymphoid tissue in SLE. Polyclonal overactivation of B-lymphocytes in SLE may produce anti-PTH receptor autoantibodies, which may activate PTH receptors, bind to PTH receptors and activate PTH-mimetic effects, and inhibit the expression of PTH and PTHrP, leading to hypercalcemia [3]. Moreover, proinflammatory cytokines such as tumor necrosis factor alpha (TNF alpha), transforming growth factor beta (TGF ß), interleukins IL-1, IL-2 and IL-6, prostaglandins E2, and granulocyte-macrophage colony stimulating factors GM-CSF) are elevated during active SLE phases and could have a direct influence on bone turnover and may stimulate osteoclast bone resorption and lead to hypercalcemia [1-3,7]. Hypercalcemia may represent a marker of disease activity [6]. However, hypercalcemia did not occur in all active SLE patients [3,6]. Patients with SLE may have hypercalcemia, due to secondary or tertiary hyperparathyroidism due to chronic renal failure related to lupus nephritis [2].

The association of SLE with primary hyperparathyroidism is very rare. First case published in 1998 with subnormal calcium serum level (104 g/l) [8], and among the 18 cases of SLE and hypercalcemia, only 5 cases was associated to Primary hyperparathyroidism [2,3,7-9]. The PHP was related to parathyroid adenoma in 4 cases [2,7-9], parathyroid cyst in one case [10] and parathyroid hyperplasia in one case [3]. The exact etiology of primary hyperthyroidism in SLE is not known. The underlying pathophysiology was supposed to be either a common genetic predisposition or the extension of the autoimmune process to the parathyroid glands, which is still under debate [3]. The lack of evidence in literature supporting the possibility of an autoimmune mechanism in the genesis of parathyroid adenoma and considering the relative frequency of these two conditions in middle-aged women, pleads for fortuitous coexistence [2,7,8]. Our patient had an active SLE with arthritis, lupus nephritis and antiphospholipid syndrome complicated by pulmonary embolism. She did not have any clinic symptoms of hypercalcemia. She did not have a renal failure but it was important to make a complete assessment to check all situations that could cause or make worse kidney function. This hypercalcemia has been fully investigated. The discovery of primary hyperparathyroidism was concomitant to the diagnosis of SLE. This association has important clinical implications. Therapeutical strategy is completely different. Parathyroid adenoma is typically treated by surgical removal which leads to complete resolution of hypercalcemia, but also provides a kidney protection especially if associated to lupus nephritis. So earlier detection of PHP might have prevented severe complications and significant morbidity.

**Patient perspective:** patient did not report serious side effects of corticosteroids or the surgery that she had. Her systemic lupus was calm. She has a good quality of life.

## Conclusion

Primary hyperparathyroidism and SLE are two serious diseases. Their association is exceptional but it could make their management more complicated. There is no evidence for potential pathogenic association between PHP and SLE. However, the recognition of this association is very important because of therapeutic and prognostic impact. It is important that physicians remember that PHP still continues to be the most common cause of hypercalcemia. Hence, when hypercalcemia presents in autoimmune conditions, such as SLE, it is prudent to do full investigations and to perform etiology to determine the most appropriate treatment for avoiding potential complications.
